# Case Report: Kampo Medicine for Non-tuberculous Mycobacterium Pulmonary Disease

**DOI:** 10.3389/fnut.2021.761934

**Published:** 2021-11-04

**Authors:** Tomoko Suzuki, Kazushi Uneda, Ryutaro Aoyagi, Takehito Kobayashi, Tadamichi Mitsuma, Hidetomo Nakamoto

**Affiliations:** ^1^Department of General Internal Medicine, Saitama Medical University, Saitama, Japan; ^2^Department of Infectious Disease and Pulmonary Medicine, Aizu Medical Center, Fukushima Medical University, Fukushima, Japan; ^3^Department of Kampo Medicine, Aizu Medical Center, Fukushima Medical University, Fukushima, Japan

**Keywords:** non-tuberculous mycobacterium-pulmonary disease (NTM-PD), Kampo medicine, hozai, bukuryoshigyakuto, hochuekkito

## Abstract

**Background:** While the number of pulmonary tuberculosis cases has decreased, increase in non-tuberculous mycobacterium pulmonary disease (NTM-PD) is a global problem. Guideline-based therapy for NTM-PD sometimes causes complications that prevent treatment completion, and there are many cases of relapse even if the treatment can be completed. In addition to antibacterial treatment, care of host risk factors, such as aging, lean physique and immunosuppressive state, is also very important for the management of NTM-PD. In Japan, Kampo medicine, a traditional Japanese herbal formulation, used alone or in combination with standard multidrug therapy for NTM-PD, has been found to be effective for such cases.

**Case Presentation:** A 77-year-old lean woman had been diagnosed with *Mycobacterium intracellulare* pulmonary infection 6 years earlier, and had received the standard multidrug treatment 5 years later at a former hospital due to worsening of her symptoms of cough, breathlessness and hemoptysis. However, the treatment was discontinued within a year due to the development of adverse events. She refused the guideline-based antibacterial treatment, and asked for Kampo medicine instead. Bukuryoshigyakuto was subsequently prescribed, which led to cough and sputum, especially hemosputum, being well controlled. With 3 years of Kampo medicine treatment, she gained weight and her hemosputum disappeared. High-resolution computed tomography images showed improvement in her lung condition, and her sputum smear culture was negative for acid-fast bacillus.

**Conclusion:** Various kinds of Kampo medicines have been used empirically for NTM-PD in Japan. A literature review from 1992 to 2020 showed that hozais, in particular, seem to be key drugs for the treatment of host NTM-PD risk factors. Kampo medicines can contribute to comprehensive treatment for NTM-PD management that does not rely solely on antibacterial drugs.

## Introduction

Non-tuberculous mycobacteria (NTM) are ubiquitous environmental organisms that live in soil and natural water sources (1). NTM commonly cause pulmonary disease (NTM-PD), which is sometimes intractable and difficult to manage (1, 2). The incidence rate of NTM-PD in Japan in 2014 was 14.7 per 100,000 people, representing an increase of 2.6 times compared to the results of a 2007 survey (3). Although basic epidemiological data are lacking in most regions, the increase in NTM-PD cases seems to have become a global problem (4–6). Over 180 NTM species have been discovered to date, only some of which are reported to cause pulmonary disease (1). The most commonly isolated species are the *Mycobacterium avium-intracellulare* complex (MAC) and *M. abscessus* complex (*M. abscessus* subsp. *abscessus*,*M. abscessus* subsp. *massiliense*,*M. abscessus* subsp. *bolletii*) (1), and a recent study in Japan showed that MAC accounts for nearly 90% of all NTM-PD cases (3).

Standard treatment regimens for NTM-PD include recommended combinations of drugs, such as macrolides and others, which need to be continued for a long period at least 1 year after culture negative was confirmed (2). This leads to the risk of treatment non-compliance; additionally, there are some cases in which treatment cannot be completed due to the occurrence of toxic adverse events (7). It was previously reported that even when treatment is undertaken in line with guidelines, treatment success rates for MAC range over 32–65% (8). Further, there are some cases that develop refractory disease with high mortality and morbidity (1). New therapeutic strategies, such as amikacin liposome inhalation, are now recommended for refractory NTM-PD cases (9), although the therapeutic options remain limited. The management of NTM-PD is therefore thought to require a holistic and multidisciplinary strategy, and not just antibiotic treatment, to achieve better outcomes (10).

Kampo medicine is a traditional Japanese herbal medicine system that originated from traditional Chinese medicine. Kampo medicines were earlier taken as a decoction. After the medicines began being supplied in dried extract form instead of the decoction in 1976, the number of people who took Kampo medicines rapidly increased in Japan. Currently, there are 148 formulas and about 150 individual herbs covered by the Japanese insurance program. Kampo medicines are traditionally used for a wide range of symptoms and diseases. The symptoms treated by Kampo medicines vary widely, and include neuralgia, arthralgia, chronic headache, shoulder stiffness, frailty and sensitivity to cold. The diseases for which they are prescribed are also diverse, and Kampo medicines are also used for respiratory diseases such as asthma (11), chronic obstructive pulmonary disease (COPD) (12) and MAC (13). Hochuekkito, a Kampo medicine, is used as an adjunct to conventional treatment for general malaise, appetite loss and physical exhaustion, and a pilot open-label quasi-randomized controlled study used the medicine in patients with progressive pulmonary MAC disease despite standard antibiotic therapy over 1 year, who were persistently culture-positive or intolerant to antibiotic therapy (13). In Japan, although guideline-based treatments have been prioritized, Kampo medicine is considered for refractory cases and cases in which it is difficult to continue antibiotic therapy due to the development of adverse effects to drugs. Therefore, various kinds of Kampo medicines have been used for NTM-PD, such as hochuekkito, ninjinyoeito, saikanto, and chikuyosekkoto (14). Above all, hochuekkito and ninjinyoeito are thought to have contributed as “hozai”s, i.e., tonic formulas, that strengthen the body and restore depleted qi and “blood” (used here as a term of traditional medicine and described later), which is one of the key principles of Kampo medicine (15). This concept is similar to the principle of addressing host risk factors of NTM-PD, such as aging and the associated frailty and immunosuppression. Another Kampo medicine that is considered as a hozai is bukuryoshigyakuto, which consists of *Ginseng radix, Aconiti tuber, Glycyrrhizae radix, Poria cocos* and *Zingiber siccatum*.

Here, we present a case of NTM-PD that was treated with bukuryoshigyakuto. Additionally, we researched and summarized previous similar reports to clarify the usefulness of Kampo medicines for NTM-PD.

## Case Description

**Figure 1:**
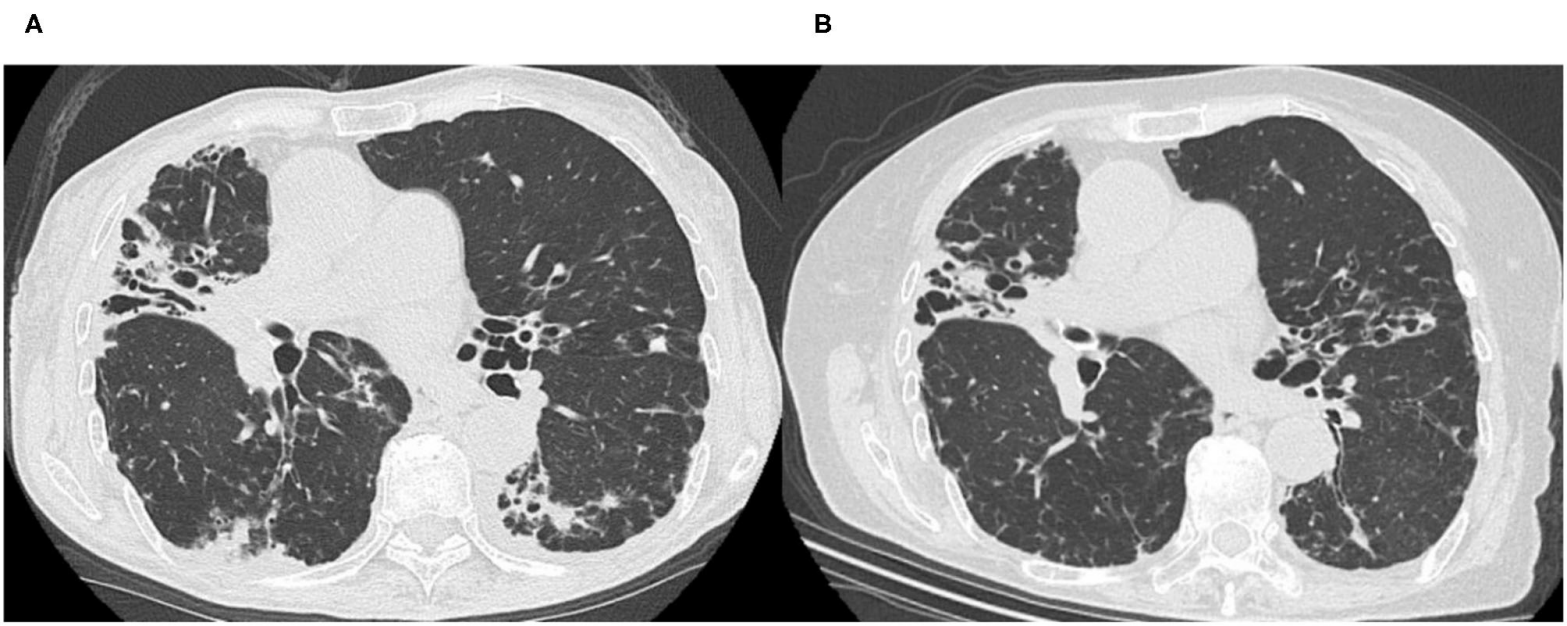
High-resolution computed tomography images before **(A)** and after **(B)** bukuryoshigyakuto treatment. **(A)** The image taken at the first visit at our hospital in 2015 showed middle lobe and lingular segment predominant bronchiectasis, bilateral centrilobular lesions, and dorsal predominant bronchiolitis. **(B)** In 2018, bilateral centrilobular lesions, consolidation, and dorsal predominant bronchiolitis lesions were improved, though the middle lobe and lingular segment bronchiectasis had no change.

A 77-year-old woman had been diagnosed with *Mycobacterium intracellulare* infection at another hospital 6 years earlier. Her symptoms of cough, breathlessness and hemoptysis worsened over 5 years without standard treatment for NTM, and combination drug therapy with macrolides (600 mg clarithromycin, 450 mg rifampicin and 750 mg ethambutol) was started 5 years after her initial diagnosis. A few months to a year later, she gradually developed adverse effects to drug therapy, such as ambulation difficulty due to numbness in her legs, weight loss and visual impairment, with persistence of cough and bloody phlegm. Hence, she decided to seek alternative treatment and was referred to our hospital. She did not have any other medical or relevant family history. She never smoked or drank alcohol. At her initial visit to us, she was 144.6 cm tall, weighed 30.0 kg, and was remarkably emaciated. She came to us in a wheelchair. Although her percutaneous oxygen (SpO_2_) saturation was 96% at rest, she could not have a long conversation, and could not even adopt the supine position, because of coughing with production of a large amount of sputum and breathlessness. Coarse crackles were heard on auscultation. A high-resolution computed tomography (HRCT) scan performed at the first consultation showed bronchiectasis that was predominantly in the middle lobe and lingular segment, bilateral centrilobular lesions and dorsal predominant bronchiolitis (Figure 1A). Her laboratory data were as follows: white blood cell count: 5,380 /μl; neutrophils: 4,180 /μl; lymphocytes: 678 /μl; C reactive protein level: 0.19 mg/dl; albumin: 3.6 g/dl; and hemoglobin: 10.8 g/dl (Table 1). According to the case notes of her previous doctor, *M. intracellulare* had been identified in her sputum, although her sputum was acid-fast bacillus (AFB) smear-negative and culture-negative at the time of her initial visit to our hospital. We tried to recommend guideline-based treatment in view of the fact that her AFB status had become negative with standard treatment for about 1 year while considering her previous adverse events to therapy. However, the patient and her family stubbornly refused antibiotic-based treatment and preferred treatment with Kampo medicines. She had remarkable emaciation, with a body mass index (BMI) of 14.4 kg/m^2^ and poor nutritional status, and she was totally exhausted due to persistent respiratory symptoms and adverse effects of therapy. She also suffered from serious appetite loss and insomnia associated with symptoms of NTM-PD. Kampo evaluation showed that her limbs were cold, pulse was weak, and abdominal strength was weak. We thus prescribed bukuryoshigyakuto based on diagnosis of yin and deficiency patterns. Hence, a bukuryoshigyakuto (*Panax ginseng* 1g, *Aconiti tuber* 1g, *Glycyrrhizae radix* 2g, *Poria cocos* 5g and *Zingiber siccatum* 2g) decoction (to infuse a total of 11 g of each herb with 400 ml of water for 30–40 min to make 200 ml, and to take in two divided doses) was started after a medical examination based on Kampo principles, while discontinuing the current treatment with clarithromycin, rifampicin and ethambutol. Within a few months, her eyesight improved, her gait disturbance also improved, and she no longer needed to use a wheelchair. Her appetite returned, and her hemoptysis and sputum gradually decreased. During the course of her treatment, she stopped taking bukuryoshigyakuto for a while due to the complexity of the decoction. She again recognized an increase in the hemoptysis and restarted the medication, and since then has never stopped it. Her HRCT image findings also improved. Although the middle lobe and lingular segment bronchiectasis showed no changes, bilateral centrilobular lesions and dorsal predominant bronchiolitis improved significantly (Figure 1B). Her body weight increased from 30 to 42 kg within 3 years after starting bukuryoshigyakuto. Her laboratory data also improved, to a neutrophil count of 2,880 /μl, lymphocytes of 1,063 /μl, albumin level of 3.9 g/dl and hemoglobin level of 11.4 g/dl (Table 1). Her shortness of breath, hemoptysis and sputum also finally improved, although she had some residual cough and slight numbness in her legs. Currently, continuing outpatient visits and bukuryoshigyakuto therapy have maintained her AFB smear and culture negative status.

**Table 1:** Laboratory data before and after bukuryoshigyakuto treatment.

	**At the first visit**	**3 years later**
Body weight (kg)	30.0	42.0
WBC (/ml)	5,380	4,600
Neutrophils (/μl)	4,180	2,880
Lymphocytes (/μl)	678	1063
CRP (mg/dl)	0.19	0.07
Total protein (g/dl)	8.0	7.9
Albumin (g/dl)	3.6	3.9
Hemoglobin (g/dl)	10.8	11.4

*It compared the body weight and the main laboratory data before and 3 years after bukuryoshigyakuto. WBC, white blood cell; CRP, c-reactive protein*.

## Discussion

The current report presents bukuryoshigyakuto treatment of an NTM-PD patient who discontinued guideline-based antibiotic therapy due to adverse events. As mentioned by Ali, management of NTM-PD, which is a debilitating, often refractory, progressive lung disease, therapy for which must be customized beyond antimicrobials to encompass various kinds of medical wisdom (10). In Japan, Kampo medicines are occasionally considered in NTM-PD cases with cough and expectoration who cannot continue standard therapy due to adverse events or in whom standard therapy is not expected to have adequate effect. The purpose of this report was to introduce the usefulness of hozais, which restore depleted qi and blood^[TM1]^, which is one of the main principles behind Kampo medicine, including bukuryoshigyakuto for NTM-PD. The International classification of Diseases 11th Revision i.e., ICD-11 now included traditional medicine in chapter 26, and [TM1] refers to Traditional Medicine conditions -Module I (16). The [TM1] designation is used for traditional medicine diagnostic category in order to be clearly distinguishable from conventional medicine concepts.

In general, some natural compounds, including Kampo medicines, have the potential to upregulate host immunity, and it is expected that this effect, rather than their bactericidal effects, is useful in the treatment of NTM (17). In fact, Chinese herbal medicines containing *Astragalus membranaceus, Radix Scutellariae, Radix Stemonae, Rhizoma Salviae Miltiorrhizae* and *Radix Euphorbiae Fischerianae* were used as adjuvant treatment to chemotherapy for multidrug-resistant tuberculosis from regard of improving immune function (18). The host risk factors for NTM-PD are well identified. Structural lung diseases, such as bronchiectasis, COPD and interstitial lung disease, are known to predispose an individual to developing NTM-PD (19–21). The onset of NTM-PD has also been recognized in persons with no previously diagnosed underlying risk factors, the so-called “Lady Windermere” syndrome that was named after a character in an Oscar Wilde novel (22, 23). The majority of subjects are female, post-menopausal, taller and thinner than average, and their susceptibility is related to hormonal factors, connective tissue abnormalities and low adiposity (24). Elderly age is also a pivotal risk factor for NTM-PD (1), and actually, the incidence of NTM-PD has increased in the super-aging society of Japan (25). Furthermore, frequent use of immunosuppressant medications, such as corticosteroids and biological therapy for collagen diseases such as rheumatoid arthritis, is also spurring an increase in NTM-PD (26). These host risk factors can sometimes overlap; for example, an old thin woman with rheumatoid arthritis treated with biological immunosuppressants might represent an ideal candidate for development of NTM-PD. The role of hozai therapy is to improve the host risk factors of NTM-PD.

Hozais, such as hochuekkito and ninjinyoeito, include a group of formulas that supposedly invigorate patients who have lost physical and mental energy due to various reasons (15), such as patients with cancer, refractory inflammatory diseases and chronic infectious diseases who are mentally and physically exhausted. NTM-PD and COPD are typical examples of these diseases, and the patients often experience an impaired quality of life (QOL) due to persistent respiratory and depressive symptoms (27, 28). In this regard, hozais could be very useful for supporting the patients' QOL. Hochuekkito is known to have immunomodulating effects by increasing serum interferon-gamma levels (29–31), and it is thought to be useful for infectious and inflammatory diseases (32). The therapeutic effect of hochuekkito on MAC is a good example (13). Enomoto et al. showed that hochuekkito is an effective adjunct to conventional therapy in patients with progressive NTM-PD (13). In their study, although none of the patients achieved sputum conversion, the number of colonies in sputum generally remained stable in the Hochuekkito group, while it tended to increase in the control group (13). Further, the hochuekkito group tended to have an increase in body weight and serum albumin levels compared with their respective values in the control group (13). Ninjinyoeito, which is another hozai for such as frailty (33), has also been used for NTM-PD. Nogami et al. reported the efficacy of ninjinyoeito in a patient with NTM-PD due to *Mycobacterium fortuitum*, who did not improve despite receiving guideline-based therapy for 2 years (34). His symptoms, such as cough, hemoptysis and general malaise, gradually improved with ninjinyoeito therapy, and 10 months later his sputum converted to smear negative (34).

In the present report, bukuryoshigyakuto was used for NTM-PD. This medicine is used in patients with yin and deficiency patterns. Bukuryoshigyakuto is also considered a hozai, and bukuryoshigyakuto-sho is recognized more deficiency than hochuekkito-sho or ninjinyoeito-sho, where “Sho” is the diagnosis of the patient's signs and symptoms comprehensively based on theories of Kampo medicine (35). Her general fatigue and appetite loss due to NTM-PD are considered good indications for hochuekkito and ninjinyoeito. However, her condition with emaciation, coldness of her limbs and weakness of her pulse was thought to be in yin and deficiency patterns, and to be more suitable for bukuryoshigyakuto than hochuekkito and ninjinyoeito. With this treatment, her appetite recovered and the cold sensation in her limbs gradually improved. Subsequently, her respiratory symptoms drastically decreased, and sputum culture remained AFB negative. According to tests for AFB, although previous treatment with antibiotics seemed to have been effective against *M. intracellulare*, it did not improve her QOL.

Bukuryoshigyakuto consists of *Panax ginseng, Aconiti tuber, Glycyrrhizae radix, Poria cocos* and *Zingiber siccatum*. Bukuryoshigyakuto is based on shigyakuto, which consists of *Aconiti tuber, Glycyrrhizae radix* and *Zingiber siccatum*. Shigyakuto is also used for patients with a yin deficiency pattern. Zang et al. reported that shigyakuto had protective effects, improving the microcirculatory disturbances induced by endotoxins in rat mesentery (36), and it could be said that improvement of microcirculatory disturbance is one of the main effects of drugs used for the treatment of a yin deficiency pattern. The effect might be associated with “ompo”, which is a method of treating deficiencies associated with cold^[TM1]^ patterns using warming-tonifying formulas. Shigyakuto has also been shown to have antiviral activity in mice infected with herpes simplex virus type 1 through the activation of CD8+ T cells (37). *Zingiber siccatum* is a pivotal constituent herb of both shigyakuto and bukuryoshigyakuto. 6-Shogaol, one of the components of *Zingiber siccatum*, was recently reported to have antibiofilm activity against *Candida albicans* (38). NTM is an environmental organism inhabiting soil and water that also forms a biofilm (39). Although there are differences between fungi and acid-fast bacilli, 6-shogaol might be effective in inhibiting biofilm formation.

Bukuryoshigyakuto also includes Ginseng radix, which is also a component of hochuekkito and ninjinyoeito. Kampo formulas containing *Ginseng radix* and *Astragali radix* are called “Jingizai”, and they are typical hozais like hochuekkito and ninjinyoeito. *Ginseng radix* (*Panax ginseng*), in particular, has been widely used in Kampo formulas. The clinical efficacy of *Panax ginseng* was well described in various kinds of studies (40). In a meta-analysis of 12 randomized controlled trials, Bach et al. reported the efficacy of ginseng supplements in alleviating fatigue (41). Another study showed that *Panax ginseng* improved respiratory muscle strength and lung function in COPD patients (42). It was also reported that Chinese herbal medicines including ginseng improved the results of 6-min walking tests in stable COPD patients (43). Further, ginsenoside Rg1, one of the saponin groups present in *Panax ginseng*, stimulates the proliferation of lymphocytes, and ginsenoside might enhance cellular immune function (44).

We reviewed the literature using PubMed and Ichushi-Web, which is a database of the Japanese Medical Abstracts Society, to identify articles which Kampo medicines were used for NTM-PD published till now. Although we and other respiratory clinicians do use hozais for NTM-PD patients on a daily basis, unfortunately, there are less articles than we expected. This limited number of patients could also be due to the fact that clinicians might not report their experiences with medicines that do not have global recognition. We performed a comparison of data before and after administration of Kampo medicines by hozai in previous cases of NTM-PD. As shown in Table 2, hochuekkito (13, 45), ninjinyoeito (34) and bukuryoshigyakuto at least seemed to increase body weight and albumin level. It is well known that chronic inflammatory pulmonary diseases result in weight loss and poor nutritional status (12). Hozais could be useful by preventing these symptoms.

**Table 2:** Comparison of previously reported data pre- and post- Kampo medicine treatment by hozai.

	**Age (year)**	**Sex**	**Add on/alone**	**Administration period of Kampo medicine (month)**	**Changes in parameter with treatment**					
					**BW (kg)**	**Alb (g/dl)**	**Hb (g/dl)**	**Ly (/μl)**	**Neu (/μl)**	**CRP (mg/dl)**
**Hochuekkito**										
Ashino and Hattori (45)	80	F	add on	12	+1	+0.3	+1.1	+405	−1,263	−1.4
Enomoto et al. (13)	70^*^	F7:M2	add on	6	+0.4^*^	+0.2^*^				+0.06^*^
**Ninjinyoeito**										
Nogami et al. (34)	72	M	alone	15	+2		+1.1			
**Bukmyoslugyaku**	77	F	alone	36	+12	+0.3	+0.6	+385	−1,300	−0.12

*Body weight, albumin, hemoglobin, lymphocytes, neutrophils, and c-reactive protein were compared. In second line, hochuekkito group*,

* *showed median quoted from Enomoto et al. (13). BW, body weight; Alb, albumin; Hb, hemoglobin; Ly, lymphocyte; Neu, neutrophil; CRP, c-reactive protein*.

Our study has some limitations in terms of clarification of the usefulness of Kampo medicines for NTM-PD. The current study is just an introduction to cases of use of Kampo medicine for NTM-PD. We admit that many doctors use Kampo medicines in their daily practice, although there are very few case reports describing this. Even if there are case reports, the data are not standardized, making them difficult to compare and summarize. Hence, it is necessary to conduct a nationwide multicenter survey to understand the use of Kampo medicines for NTM-PD, including a retrospective observation study. For further investigation, a randomized controlled study will be the next step to investigating the usefulness of Kampo medicines for NTM-PD.

## Data Availability Statement

The original contributions presented in the study are included in the article/supplementary material, further inquiries can be directed to the corresponding author.

## Author Contributions

TS, RA, TK, and HN designed the study. TS and KU collected the data. TS, TK, and KU analyzed the data. TM advised about Kampo medicines. TS and HN wrote the manuscript. All authors read and approved the final manuscript.

## Conflict of Interest

The authors declare that the research was conducted in the absence of any commercial or financial relationships that could be construed as a potential conflict of interest.

## Publisher's Note

All claims expressed in this article are solely those of the authors and do not necessarily represent those of their affiliated organizations, or those of the publisher, the editors and the reviewers. Any product that may be evaluated in this article, or claim that may be made by its manufacturer, is not guaranteed or endorsed by the publisher.

## Abbreviations

AFB: acid-fast bacillus
BMI: body mass index
COPD: chronic obstructive pulmonary disease
HRCT: high-resolution computed tomography
MAC: *Mycobacterium avium-intracellulare* complex
NTM: nontuberculous mycobacteria
NTM-PD: NTM commonly causes pulmonary disease
QOL: quality of life
SpO_2_: percutaneous oxygen
[TM1]: [traditional medicine module 1].
